# Operational Status of Isolation Rooms in Emergency Departments and Patient Concentration in Higher-Level Emergency Departments in Daegu Metropolitan City and Neighboring Provinces, South Korea, during the COVID-19 Pandemic

**DOI:** 10.3390/ijerph20043113

**Published:** 2023-02-10

**Authors:** Heonjoo Kim, Hansol Chung

**Affiliations:** Department of Emergency Medicine, Yeungnam University College of Medicine, Daegu 42415, Republic of Korea

**Keywords:** isolation room, COVID-19, emergency department, nosocomial transmissions

## Abstract

Background: In a pandemic situation such as the one of the COVID-19 pandemic, nosocomial transmissions attempted to be prevented by initially classifying them in triage. Therefore, emergency departments (EDs) installed isolation rooms at their entrance. Additionally, a system for pre-emptive quarantine at the triage stage was established nationwide for patients with COVID-19-related symptoms. Methods: Data were retrospectively collected from 28,609 patients who visited the regional emergency medical center of Yeungnam University Hospital in Daegu Metropolitan City in 2021. The study population was divided into experimental and control groups comprising patients with and without COVID-19-related symptoms, respectively. The difference in the percentage of patients visiting from outside the city was investigated between the two groups. The critically ill patient (CP) ratio was analyzed in the experimental group to verify the appropriateness of visiting a higher-level ED and was further divided into sub-regions to determine their reason for visiting an ED beyond their residential region. Results: Most lower-level EDs did not have isolation rooms. About 20.1% and 17.3% of patients in the experimental and control groups visited a higher-level ED with an isolation room beyond their residential region, respectively. The absence of an isolation room in the ED in their residential region was one reason for traveling beyond their residential region, with an odds ratio of 4.44 (95% confidence interval: 0.53–8.35). Conclusion: In the process of implementing the “pre-emptive quarantine” system, it was revealed that the cooperation of the lower-level EDs was not effective during the implementation of the “pre-emptive quarantine” system. Consequently, a higher number of patients with COVID-19-related symptoms had to locate an ED with an isolation room and travel a longer distance than general patients. The participation of more EDs is required.

## 1. Background

The current COVID-19 pandemic has demonstrated that emergency departments (EDs) are at the forefront of hospital and community-based care during viral respiratory disease outbreaks. Therefore, it is essential to prevent nosocomial transmissions by properly classifying them in triage at the initial stage, since various patients, including infected, non-infected, and trauma patients, visit the ED. The first COVID-19 patient was reported in Wuhan in December 2019, while the first patient in South Korea was reported in January 2020. Before the initial revision of the triage in the ED, nosocomial transmissions occurred when an infected person visited the ED, which caused medical confusion. As a countermeasure, in the case of COVID-19-related symptoms, an isolation room was installed at the EDs entrance. Additionally, a system for pre-emptive quarantine at the triage stage was established in South Korea and other countries [1,2,3,4,5,6]. These countermeasures significantly stabilized the daily operations of the ED.

EDs responses to COVID-19 varied across countries. The “pre-emptive quarantine”, which was the basis of this study, was implemented in much the same way globally [1,2,3,4,5,6] and is a viable solution for another pandemic. However, problems were revealed in the process of implementing the quarantine method. One problem was the uncertainty of small- and medium-sized hospitals with EDs concerning the installation of isolation rooms, which caused overcrowding of higher-level EDs. Even patients with a mild fever were rushed to tertiary hospitals, including regional emergency medical centers. According to a national report from the Ministry of Health and Welfare, 2959 of the 173,962 patients who were reported to 119 with a fever were refused ED medical care at least once during the COVID-19 pandemic between January 2020 and August 2021. Although these 2959 patients accounted for only 1.7% of the total, the refusal of ED medical care is quite rare. Therefore, it has become a point of debate in the parliamentary audit [7,8]. Even though isolation rooms have been expanded several times, mainly in higher-level EDs, the problem has not been resolved.

Previous studies that aimed at preventing nosocomial transmissions through the ED have mainly proposed new systems, such as the resetting of the isolation method, revision of the ED at triage, restrictions on patient visits, and telemedicine [9,10,11,12,13,14]. It has been three years since the COVID-19 pandemic, so it is time to examine the problems identified in the new systems that have been implemented thus far and develop countermeasures. However, there is a paucity of related studies. Therefore, the present study aimed to analyze whether patients with COVID-19-related symptoms had difficulty choosing an ED near their residence due to the “pre-emptive quarantine” system and determine counter measures.

## 2. Methods

### 2.1. Study Population and Data Collection

**Figure 1:**
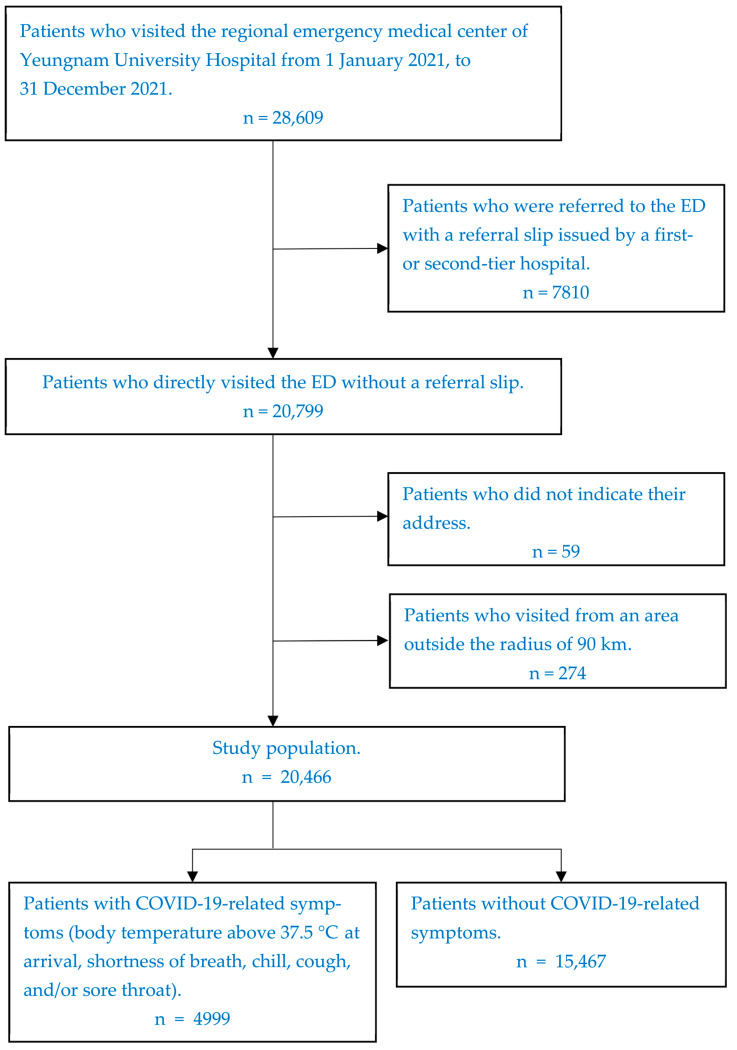
Flow diagram of the study population. (ED, emergency department; COVID-19, coronavirus disease 2019).

This study retrospectively collected data from 28,609 patients who visited the regional emergency medical center of Yeungnam University Hospital in Daegu Metropolitan City from 1 January 2021 to 31 December 2021. Data were extracted from the electronic medical record system of Yeungnam University Hospital in South Korea and were completely anonymized during the extraction process. However, 7810 patients who were transferred to the ED with a referral slip issued by a first- or second-tier hospital were excluded because they were an appropriate visit according to the medical system and could not be considered as a cause of overcrowding in the higher-level ED. Fifty-nine patients whose residential areas were not specified were excluded. Additionally, patients who visited the ED from a distance that could not be considered a neighboring province, most of whom fell sick suddenly while traveling for personal or business reasons, were excluded because they did not match the study’s purpose. The distance criterion was set within a radius of 90 km, leading to the exclusion of 274 patients. The final analysis included 20,466 patients. Among them, 4999 patients with COVID-19-related symptoms were assigned to the experimental group, while 15,467 patients without COVID-19-related symptoms were included in the control group. COVID-19-related symptoms refer to a fever of 37.5 °C or higher, shortness of breath, chills, cough, and a sore throat (Figure 1). Yeungnam University Hospital, where the research was conducted, is located in the geographical center of the Daegu Metropolitan City. The year 2021 was chosen as the study period because the pre-emptive quarantine method of caring for patients with COVID-19-related symptoms in the isolation room until they tested negative in a COVID-PCR or antigen test was standardized in 2021.

### 2.2. Study Design

Among the study population, patients with COVID-19-related symptoms were included in the experimental group, while other patients were included in the control group. The difference in the proportion of patients who traveled a long distance for medical care despite living outside Daegu Metropolitan City was analyzed between the two groups. The experimental group underwent several additional analyses. First, the critically ill patient (CP) ratio was calculated to verify the appropriateness of visiting a higher-level ED. Second, the “Outside the Metropolitan City visits” group was further subdivided into “A-n” or “B-n” (areas less than 45 km away from the Daegu Metropolitan City are marked as “A,” and those farther than 45 km are marked as “B”), and the “distance” from the Daegu Metropolitan City by region, “the number of patient visits to our hospital relative to the residents in each region”, “operational status of the isolation room”, and the “number of EDs” were set as the influencing factors. This allowed for an analysis of the factors affecting the movement of patients with COVID-19-related symptoms over long distances to seek medical care. The number of patient visits relative to the residents by region was based on 2021 demographic data points [15]. It was expressed as the “number of patient visits per 10,000 residents (NPVR)”, and the “distance” was measured based on our hospital and the distance from zero milestones in each region. It was measured as the straight-line distance between two points using the map service provided by the National Geographic Information Institute.

### 2.3. Critically Ill Patients (CPs)

In this study, the patients who visited Yeungnam University Hospital even though it was located outside of their residential region were divided into critically ill patients (CP), who required treatment from a higher-level ED, and non-critically ill patients (NCP), who were able to receive sufficient treatment at the local ED. There are various methods for defining the severity of a patient’s condition in the ED, such as the method based on the assessment of vital signs and mental state, the method based on the need for medical resources, and the method based on the diagnosis. This study used the “Korean Triage and Acuity Scale” (KTAS) method to evaluate the severity of the patient’s initial classification and the “Severe illness code” (a diagnosis of severe emergency disease) to evaluate the severity after the termination of treatment. Therefore, two methods were used to define a seriously ill patient [16].

The KTAS is a five-level triage tool developed based on the Canadian Triage and Acuity Scale. It determines a patient’s priority for treatment by providing a possible waiting time for treatment based on their main symptoms, vital signs, and intensity of pain. The lower the level, the more urgent the patient’s condition. Level 1 patients require emergent treatment and have life- or limb-threatening conditions. Symptoms, such as cardiac arrest or apnea, are typical examples. Level 2 patients face potential threats to life, limbs, and body functions, and require immediate treatment. These include patients with suspected myocardial infarction or stroke. For Level 3 patients, the possibility of developing a condition that requires treatment should be considered. They should receive treatment or a re-examination within 1–2 h after considering their age, pain level, and possibility of deterioration and complications. Level 4–5 patients do not require urgent treatment. These patients may have chronic disorders that are less likely to deteriorate. In this study, patients with KTAS levels of 1 or 2 were classified as CPs [17,18,19]. A “severe illness code” was assigned to each patient after the final diagnosis at the termination of treatment.

A “severe illness code” is defined as a disease or trauma with a high risk of death and whose acute treatment has a close influence on the patient’s prognosis (Table S1). It consists of 28 disease categories with a survivor risk ratio of ≤0.95. It is recommended that patients with this diagnosis be treated in a higher-level ED. Furthermore, it is used as an evaluation factor to analyze the functionality and timeliness of emergency medical institutions [20].

The patient was classified as a CP even if only one of the above criteria were met.

### 2.4. Level Classification of EDs in South Korea

According to the Emergency Medical Act, emergency medical institutions in South Korea are divided into regional emergency medical centers (Level 1), local emergency medical centers (Level 2), local emergency medical agencies (Level 3), and other emergency medical facilities (Level 4) that operate independently without national designation, according to the size, facility, and level of manpower. Most emergency rooms in South Korea surpass the minimum standards. The Ministry of Health and Welfare designates the regional emergency medical centers. They are selected from tertiary general hospitals with more than 300 beds and 20 specialties and are recommended to focus on critically ill patients. Concerning facilities, they should operate a negative-pressure isolation bed and have a CT and two ambulances mandatorily. Their manpower should include at least 5 emergency medicine specialists, 1 pediatric specialist, 25 nurses, and 1 pediatric nurse. Metropolitan city mayors and provincial governors designate local emergency medical centers from general hospitals with more than 100 beds, more than 7 specialties, an X-ray facility, at least 1 ambulance, 4 ED physicians, and 10 nurses. The mayor designates local emergency medical agencies as hospitals with more than 30 beds, 4 or more specialties, an X-ray facility, at least 1 ambulance, 2 ED physicians, and 5 nurses.

In accordance with the “Enforcement Rules of the Emergency Medical Services Act” amended in December 2020, EDs above the center level (Level 2) are required to have at least one negative-pressure isolation room or general isolation room.

### 2.5. Isolation Room and Its Operational Status in Daegu Metropolitan City and Neighboring Provinces

Data from the National Emergency Department Information System (NEDIS) were used to identify whether an ED operated in any of the neighboring provinces (Gyeongsangbuk-do, Gyeongsangnam-do, and Ulsan), including Daegu Metropolitan City. The NEDIS, an ED information network operated by the government (Ministry of Health and Welfare), is managed by the National Emergency Medical Center.

The isolation room included a negative-pressure isolation room and all areas operated as single rooms even without the negative-pressure facility. After a patient with confirmed COVID-19 was discharged, the isolation room was disinfected and ventilated before reuse.

### 2.6. Statistical Analysis

Statistical analyses were performed using IBM SPSS (version 27.0; IBM Corp., Armonk, NY, USA). Pearson’s chi-squared test was used to analyze the categorical variables. The number of EDs and patients is expressed as n (%). In the experimental group, the difference in the CP ratio between the inside and outside of the metropolitan city was expressed as n (%) and the odds ratio. The difference in the number of patients in the experimental and control groups who visited from inside and outside the metropolitan city was expressed in the same way. Univariate logistic regression analysis was performed to identify the factors affecting NPVR with NPVR as the dependent variable and the distance between the regions, the number of EDs in the region, and the operational status of the isolation room in the region as independent variables. The results of the logistic regression analysis are presented with the odds ratio and 95% confidence interval along with the *p*-value. Its significance level was set at *p* < 0.05.

## 3. Results

### 3.1. Operational Status of Isolation Rooms in Daegu Metropolitan City and Neighboring Provinces

Daegu Metropolitan City has 2 regional emergency medical centers, 4 local emergency medical centers, and 10 local emergency medical agencies. The neighboring provinces have 7 regional emergency medical centers, 13 local emergency medical centers, and 42 local emergency medical agencies. Across them, there are a total of five isolation rooms in Daegu Metropolitan City and eight in the neighboring provinces. Generally, the isolation room is operated by the regional emergency medical center in each area. Among the local emergency medical centers, only three large hospitals in Daegu Metropolitan City and one hospital in the neighboring provinces operated an isolation room. However, although these four EDs are local emergency medical centers in terms of their administrative classification, they are affiliated with university hospitals and their scale is similar to regional emergency medical centers. In conclusion, only the ED at the regional emergency medical center or the ED attached to the university hospital operated an isolation room (Table 1).

### 3.2. Comparison of the Number of Patient Visits from Outside the Metropolitan City between the Experimental and Control Groups

Table 2 shows the difference in the number of patients who visited from outside the metropolitan city between the control and experimental groups. In the control group, the proportion of patients who visited our hospital from outside the metropolitan city was 17.3%, whereas it was 20.1% in the experimental group. A significant difference was observed between the groups (*p* < 0.001).

### 3.3. Comparison of the Number of Patient Visits Inside and Outside the Metropolitan City

Table 3 shows that the number of visits by patients residing inside the metropolitan city in the entire study sample was 3995 (79.9% of the total number of visits), while the number of visits by patients residing outside the metropolitan city was 1004 (20.1% of the total). Among them, the proportion of CPs who required treatment at a regional emergency medical center was 17.4% and 20.5%, respectively, and the odds ratio was 1.23. The proportion of CPs was somewhat higher in the “Outside the metropolitan city visits” group as compared to the other group.

### 3.4. Number of Patient Visits per 10,000 Residents in Each Region Outside the Metropolitan City

Table 4 displays the subdivision of areas outside the metropolitan city into “A-n” or “B-n”, the NPVR, the distance from the region, the number of EDs in the region, and the presence of an ED operating an isolation room in the region. Data are arranged in a descending order based on the NPVR. The region with the highest NPVR is the A-6 region, which is close to the hospital under study; however, it does not have an operational isolation room. Of the 30 sub-regions, the top 13 did not have an operational isolation room.

**Figure 2:**
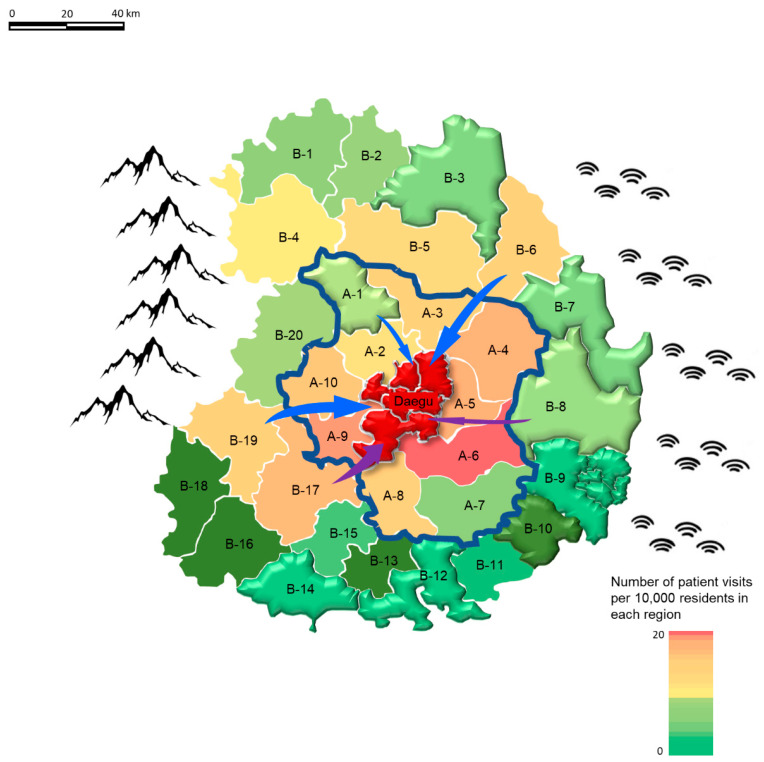
Number of patient visits per 10,000 residents in each region outside the metropolitan city. (Among the neighboring provinces, areas less than 45 km away from the Daegu Metropolitan City are marked as “A”, and those farther than 45 km are marked as “B”. Each subregion within A and B was identified by marking the letter with a number, “-n”, in clockwise order. Regions that operated at least one isolation room are shown in three dimensions.).

The results are shown in Figure 2. As shown on the map, the west and east sides are surrounded by mountains and sea, respectively. The NPVR is expressed as a regional color, with a higher value expressed in red and a lower value expressed in green. The headquarters are located in Daegu Metropolitan City represented by the red purple area in the center. It is also the largest metropolitan area in the vicinity as well as the research base. Areas that operated at least one isolation room are shown in three dimensions.

The NPVR tended to decrease if the area was farther away from the center and it operated an isolation room.

### 3.5. Factors Influencing the Number of Patient Visits per Residents

Table 5 shows the results of the univariate logistic regression analysis with the NPVR for each sub-region as the dependent variable, and the distance from our hospital, the number of EDs in the region, and the operational status of the isolation room in the region as the independent variables. The NPVR significantly increased as the distance decreased and if an isolation room was not operated by the ED closest to the patient’s residence.

## 4. Discussion

Since the COVID-19 outbreak became a pandemic, when patients with COVID-19-related symptoms visited the ED in South Korea, a COVID-19 test was conducted to prevent nosocomial transmissions. They were cared in an isolation room until the test results were negative. Therefore, EDs must be equipped with isolation rooms for the smooth functioning of medical care processes; however, lower-level EDs hesitate to operate isolation rooms because of high installation costs and a low profitability [21,22]. Isolation rooms, mainly in higher-level EDs, were expanded several times; however, the participation of lower-level EDs was passive. Consequently, whenever the number of confirmed COVID-19 cases increased, the number of isolation rooms in regional emergency medical centers increased. However, this led to a concentration of patients in a small number of higher-level EDs. Instead of installing isolation rooms in more EDs, increasing the number of isolation rooms only in the higher-level EDs caused burnout among higher-level ED workers.

Of the 4999 patients who primarily visited our hospital due to COVID-19-related symptoms, 900 patients with severe illness who required treatment at the regional emergency medical center accounted for only 20% of the total. In other words, it would have been better if the rest of the 80% of the patients had tried visiting a lower-level ED first. The CP rate in the “Outside the metropolitan city visits” group was 20.5%, which was somewhat higher than the CP rate of 17.4% among the “Inside the metropolitan city visits” group. Nevertheless, the CP rate was too low to explain the visit to the regional emergency medical center located beyond the area of their residence (Table 2). Regardless of the severity, several patients visit regional emergency medical centers for better care; however, the centers are too far for patients to be visiting for that reason alone. In Daegu Metropolitan City, in addition to the regional emergency medical centers, three local emergency medical centers operated isolation rooms. Therefore, the situation here was better. However, since few places operated isolation rooms outside the metropolitan city, it was difficult to accommodate COVID-19 patients in the area. Thus, even patients with a simple fever residing outside the region visited our hospital. This study confirmed that the non-operation of isolation rooms in the residential region was a factor that caused patients to visit distant Eds (Table 3 and Table 4). Figure 2 shows that a smaller number of patients residing in a region with an ED operating an isolation room visited Eds outside the area. For example, the distance between the base region and B-8 region is 56.2 km, and the distance from the B-17 region is 51.9 km, which are similar in distance. However, the NPVR in the B-17 region was 9.09, which was much higher than 0.99 in the B-8 region, which operates an isolation room. The A-1 region, another area that operates an isolation room, had a low NPVR of 2.19 compared to 6.02 and 5.19 in the more distant regions of the B-6 and B-19 region, respectively, despite its close distance of 36 km (Figure 2).

In a pandemic situation where there is a shortage of medical staff, it is inefficient in terms of the distribution of medical personnel for all patients with COVID-19-related symptoms to concentrate on a small number of medical staffs working at higher-level EDs. Considering the current situation, hospitals of a certain size or larger should fulfil their minimum social responsibilities in return for the income earned from the local community. A negative-pressure isolation room should be installed. If that is an impractical solution for commercial hospitals, a general isolation room without a negative-pressure facility might be a viable alternative. If this is also difficult, the “in-personal vehicle care method”, which treats the patient’s personal vehicle as an independent isolation room, can be used [2].

A sustainable and effective alternative would be to encourage more medical staff to actively participate in medical care by not only increasing the number of isolation rooms expressed by the number of beds but also by increasing the available manpower. If lower-level EDs could increase their contribution, it would provide considerable assistance in passing the current situation.

The strength of this study is that it is the first to suggest countermeasures for the weaknesses identified in the process of implementing the “pre-emptive quarantine” system. Although this study was conducted in a representative higher-level ED, the inclusion of a single center is a limitation. Future studies should utilize larger datasets.

## 5. Conclusions

Lower-level EDs did not cooperate effectively during the process of implementing the “pre-emptive quarantine” system. Consequently, a higher number of patients with COVID-19-related symptoms had to locate an ED with an isolation room and travel a longer distance than general patients. The participation of more EDs is required.

## Figures and Tables

**Table 1:** Operational status of isolation rooms by ED level and area.

Area	ED Level	Isolation Room Operation ED n (%)	Total ED n
Total	Regional emergency medical center (Level 1)	9 (100)	9
	Local emergency medical centers (Level 2)	4 (23.5)	17
	Local emergency medical agency (Level 3)	0 (0)	52
Daegu Metropolitan City	Regional emergency medical center (Level 1)	2 (100)	2
	Local emergency medical centers (Level 2)	3 (75)	4
	Local emergency medical agency (Level 3)	0 (0)	10
Neighboring provinces (outside the metropolitan city)	Regional emergency medical center (Level 1)	7 (100)	7
	Local emergency medical centers (Level 2)	1 (16.7)	13
	Local emergency medical agency (Level 3)	0 (0)	42

ED, emergency department.

**Table 2:** Comparison of the number of patient visits from outside the metropolitan city between the experimental and control groups.

	Total	Outside the Metropolitan City Visits n (%)	OR (95% CI)	*p*-Value
Patients without COVID-19-related symptoms	15,467	2664 (17.3)	Reference	*-*
Patients with COVID-19-related symptoms	4999	1004 (20.1)	1.21 (1.11–1.31)	<0.001

Values are presented as numbers (%). The difference in the proportion is expressed as OR (95% CI). OR, odds ratio; CI, confidence interval.

**Table 3:** Comparison of the number of visits by patients with COVID-19-related symptoms from inside and outside the Daegu Metropolitan City.

Variables	Total n (%)	Critical Patients n (%)	OR (95% CI)	*p*-Value
Total	4999	900 (18.0)	-	*-*
Inside the Metropolitan City visits	3995 (79.9)	694 (17.4)	Reference	*-*
Outside the Metropolitan City visits	1004 (20.1)	206 (20.5)	1.23 (1.03–1.46)	0.021

Values are presented as numbers (%). The difference in the proportion of critical patients was expressed as odds ratio (95% CI).

**Table 4:** Number of visits by patients with COVID-19-related symptoms and isolation rooms in regions outside the Daegu Metropolitan City.

Regions	NPVR	Whether the Isolation Room Is in Operation	Distance (km)	Number of ED
A-6	20.59	X	27.6	1
A-9	13.71	X	34.5	1
A-5	10.50	X	13.6	2
A-4	10.39	X	32.4	1
B-17	9.09	X	51.9	1
A-10	7.37	X	29.2	1
A-8	6.20	X	35.3	1
A-3	6.02	X	41.4	0
B-19	6.02	X	65.7	1
B-6	5.19	X	74.9	1
B-5	4.83	X	54.2	1
A-2	3.92	X	22.6	0
B-4	2.37	X	71.9	2
A-1	2.19	O	36.0	3
B-20	1.21	X	53.0	2
B-2	1.08	X	88.4	1
B-8	0.99	O	56.2	1
B-1	0.98	X	87.7	2
A-7	0.95	X	42.9	1
B-3	0.76	O	78.3	3
B-7	0.72	O	68.7	5
B-15	0.37	X	68.3	1
B-11	0.15	X	76.0	5
B-14	0.06	O	88.9	6
B-9	0.04	O	74.0	7
B-12	0.04	O	70.0	9
B-10	0	O	71.4	2
B-13	0	X	68.8	0
B-18	0	X	88.2	1
B-16	0	X	83.0	1

NPVR: number of patient visits per 10,000 residents. The regions are listed in descending order based on the NPVR. If at least one isolation room is in operation in the region, it is marked with “O”. And if no isolation room is in operation in the region, it is marked with “X”.

**Table 5:** Analysis of factors affecting the NPVR.

Variables	OR (95% CI)	*p*-Value
Not operating isolation room	4.44 (0.53–8.35)	0.027
Distance (km)	−0.16 (−0.22–−0.09)	0.0
Number of ED	−0.82 (−1.65–0.01)	0.052

## Data Availability

Data privacy regulations prohibit the deposition of individual-level data to public repositories. Furthermore, ethical approval does not cover the public sharing of data for unknown purposes. However, the corresponding author can provide the data if their use is covered by ethical approval.

## Supplementary Materials

The following supporting information can be downloaded at: https://www.mdpi.com/article/10.3390/ijerph20043113/s1, Table S1: Disease categories in the severe illness code.

## Abbreviations

COVID-19	Coronavirus disease-19
ED	Emergency department
NPVR	Number of patient visits per 10,000 residents
CP	Critically ill patients
NCP	Non-critically ill patients
NEDIS	National Emergency Department Information System
KTAS	Korean Triage and Acuity Scale
