# Supplementary material for: Operational Status of Isolation Rooms in Emergency Departments and Patient Concentration in Higher-Level Emergency Departments in Daegu Metropolitan City and Neighboring Provinces, South Korea, during the COVID-19 Pandemic
Source: Int J Environ Res Public Health. 2023 Feb 10;20(4):3113. doi: 10.3390/ijerph20043113 (PMC9961030; doi:10.3390/ijerph20043113)
Supplement: Supplementary file 1 [file ijerph-20-03113-s001.zip › ijerph-2143564-supplementary.pdf]

Table S1. Disease categories of severe illness code

No.	Disease category	KCD-8 disease code
1	Acute myocardial infarction	I210-I219
2	Acute stroke	I6300-I64
3	Intracranial hemorrhage	I610-I629
4	Subarachnoid hemorrhage	I600-I609
5	Major trauma	S0610-S0611, S0650-S0651, S0660-S0661, S0670-S0671, S0680-S0681, S0200, S0201, S0210, S0211, S0620, S0621, S0690, S0691 , S0640-S0641, S150, S250, S2600, 2601,S2680,S2681,S2690,S2691, S2710, S2711, S2720, S2721, S280, S2730, S2731, S32820-S32891, S351, S352, S353, S354, S355, S357, S359, S36100-S36112, S3670, S3671, S3680, S3681, S3770-S3771, S396, S3640, S3641, S3650, S3651, T0250-T0251, T790-T791, T71, T751<br>ICISS <= 0.90
6	Aortic dissection	I7101-I7109, I7110-I7119, I713, I715, I718
7	Hepatobiliary diseases	K8000-K8011, K8030-K8041, K8051, K810, K819, K830, K831
8	Surgical diseases	K352-K353, K631, K650-K659, K661
9	Gastrointestinal bleeding/foreign bodies	I8500-I8501, I864, I983, K920-K922, K226, K2500, K2540, K2501, K2521, K2541, K2561, K260, K262, K264, K266, T181
10	Tracheobronchial bleeding/foreign bodies	R042, R048, R049, T1740-T1799
11	Intoxication (including CO poisoning)	T360-T659
12	Peripartum diseases	O000-O009, O140-O159, O4200, O4201, O4209, O4210, O4211, O4219, O4220, O4221, O4229, O4290, O4291, O4299, O450-O459, O6000-O6039, O800-O809, O820-O829, O720-O723, O622
13	Preterm/low birth weight	P0700-P0739, P220-P229, P240-P249, P360-P369, P520-P529, P590-P599
14	Major burn	T3130-T3199, T2030-T2039, T2070-T2079, T213, T217
15	Status epilepticus	G410-G419
16	Severe infection	A830-A879, G000-G07, A227, A241, A267, A400-A409, A410-A414, A419, A427, B007, B377
17	Diabetic coma	E1000-E1018, E1100-E1118, E1300-E1318, E1400-E1418
18	PTE/DVT	I260, I269, I802
19	Arrhythmia	I441, I442, I450-I459, I472, I480-I489, I490, I495, I498, I499
20	ARDS/pulmonary edema	J80, J81, J850-J869, J9600-J9699
21	DIC	D65
22	Intussusception/intestinal obstruction	K561-K563, K565-K566
23	Amputation	S480-S489, S580-S589, S6800-S689, S780-S789, S880-S889, S980-S984, T050-T059, T060-T068, T116, T136
24	Acute kidney injury	N170-N179, E1128
25	Ophthalmic emergencies	H3300-H3309, H3310-H332, H3330-H334, H3350-H3358, H340-H349,H400, H4010-H4019, H4020-H403, H404, H405, H406, H4080-H409, H420, H428
26	Post resuscitation status	I460-I469
27	Urological emergencies	N44, N4500-N4502, N4590-N4592
28	Shock	T794, T886, T780, T805, T782, R570, R571, R572

KCD, Korean Standard Classification of Diseases; CO, carbon monoxide; PTE, pulmonary thromboembolism; DVT, deep vein thrombosis; ARDS, acute respiratory distress syndrome; DIC, disseminated intravascular coagulopathy.
